# Pre- and Post-Radioactive Iodine Therapy Hormonal Changes and Clinical Outcomes in Hyperthyroid Patients: A Retrospective Cohort Study

**DOI:** 10.12669/pjms.42.6.13855

**Published:** 2026-06

**Authors:** Arshad Hussain, Aqil Noor, Mohammad Saadullah, Bushra Haider

**Affiliations:** 1 Arshad Hussain, Professor Endocrinology, Northwest General Hospital and Research Centre, Peshawar, Pakistan; 2 Aqil Noor, Assistant Professor Endocrinology, Northwest General Hospital and Research Centre, Peshawar, Pakistan; 3 Mohammad Saadullah, Consultant Physician, Nuclear Medicine, Northwest General Hospital and Research Centre, Peshawar, Pakistan; 4 Bushra Haider, Research Officer, Northwest General Hospital and Research Centre, Peshawar, Pakistan

**Keywords:** Graves’ Disease, Hyperthyroidism, Hormone Profile, Hypothyroidism, Iodine Therapy, Radioactive

## Abstract

**Objective:**

To assess changes in thyroid hormone levels following radioactive iodine (RAI) therapy, evaluate post-treatment clinical outcomes, and identify factors associated with thyroid status among hyperthyroid patients treated at a tertiary care hospital in Peshawar.

**Methodology:**

This was a retrospective cohort study conducted at Nuclear Medicine and Endocrinology Department of Northwest General Hospital, from January 2022 to December 2024. A total of 83 adult patients with Graves’ disease, toxic adenoma, or toxic multinodular goiter who received RAI therapy were included. RAI was administered using a fixed-dose protocol based on clinical diagnosis and disease severity. Antithyroid drugs were discontinued prior to RAI as per standard institutional protocol. Baseline demographic and clinical characteristics, including age, gender, and diagnosis, were recorded. Thyroid hormone levels (Free T4, T3, and TSH) were measured at baseline and six months post-treatment. Data on thyroid gland size and TRAb levels were not routinely available and therefore not included in the analysis. Only patients with complete six-month follow-up data were analyzed. Pre- and post-treatment hormone levels were compared using the Wilcoxon Signed-Rank Test, and binomial logistic regression was used to identify predictors of thyroid status.

**Results:**

The mean age of patients was 49.76±15.2 years, predominantly female (69%). Graves’ disease was the most common diagnosis (54.8%). At six months post-RAI therapy, 44.0% developed hypothyroidism, 41.7% became euthyroid, and 14.3% remained hyperthyroid. Post-treatment thyroid status was significantly associated with underlying diagnosis (p < 0.001), while no significant associations were observed with age (p=0.060) or gender (p = 0.364). Significant reductions in Free T4 (2.19 to 1.01 ng/dl) and T3 (2.01 to 1.02 ng/dl), along with an increase in TSH (0.01 to 3.65 mIU/L), were observed following RAI therapy (p<0.001). On regression analysis, Graves’ disease was identified as a significant predictor of post-treatment hypothyroidism (adjusted OR=16.48, 95% CI: 3.14–86.58; p=0.001), whereas age, gender, toxic adenoma, and RAI dose were not statistically significant predictors.

**Conclusion:**

RAI therapy was associated with hypothyroidism as a common outcome, particularly among patients with Graves’ disease, while other clinical factors showed limited association with post-treatment thyroid status.

## INTRODUCTION

Hyperthyroidism is a condition characterized by excessive production of thyroid hormones. It has an estimated global prevalence of approximately 2.5% among adults. Overt hyperthyroidism occurs in about 0.2%–1.4% of the population, whereas subclinical hyperthyroidism is slightly more prevalent, affecting 0.7%–1.4%.^1^ In Pakistan, the prevalence of hyperthyroidism is (2.9%), higher than that reported in the United States (1.3%) and Europe (0.8%). However, direct comparisons are limited by potential variations in diagnostic criteria, population characteristics, and study methodologies.^1^ It is more prevalent in women, particularly with advancing age, among reproductive-age women, and is commonly associated with autoimmune disorders, genetic predisposition, and environmental factors. Management options include antithyroid medications, radioactive iodine therapy, and surgical intervention, with timely treatment being essential to reduce morbidity and prevent long-term complications.^2^,^3^,^4^

Graves’ disease is the leading cause of hyperthyroidism, followed by toxic nodular goitre and thyroiditis.^5^ Risk factors include autoimmune diseases, genetic predispositions, and environmental exposures such as iodine fortification, which can transiently increase the incidence of thyrotoxic nodular disease.^6^ Untreated hyperthyroidisms can lead to severe health issues, including cardiac arrhythmias, osteoporosis, and adverse pregnancy outcomes.^1^ The condition is associated with increased mortality, emphasizing the need for timely diagnosis and treatment.^1^

Antithyroid drugs (ATD) like methimazole and carbimazole, are commonly used as first-line treatments for hyperthyroidism. They work by inhibiting thyroid hormone synthesis. Studies found that ATDs can maintain normal thyroid function in a significant portion of patients, though relapse is common, especially in younger patients and those with fluctuating thyrotropin receptor antibody levels.^7^,^8^ RAI is a widely accepted treatment for hyperthyroidism, with a high success rate in achieving euthyroidism 75.6 %.^9^

A study conducted at Peshawar from 2013 to 2019 on 153 hyperthyroid patients report that 19.6% became euthyroid, 9.2% remained hyperthyroid, and 25.5% developed hypothyroidism after six months RAI therapy. The remission rate by the end of six months was 80.95%. The study reported heterogeneous outcomes following RAI therapy, with an unexpected higher rate of hypothyroidism among patients receiving lower doses (≤20 mCi). This finding contrasts with the conventional dose–response relationship and may reflect confounding by indication, where treatment allocation is influenced by disease severity and clinical characteristics. Such observations highlight the need for cautious interpretation of dose-related outcomes in non-randomized settings.^10^

Despite well-established global evidence on radioactive iodine (RAI) therapy, there is limited region-specific data from Pakistan regarding post-treatment hormonal changes and clinical outcomes. Regional variations in disease characteristics, healthcare access, and treatment practices may influence these outcomes. This study aimed to assess changes in thyroid hormone levels following RAI therapy, evaluate post-treatment clinical outcomes, and determine factors associated with thyroid status among hyperthyroid patients treated at a tertiary care hospital in Peshawar. The findings are expected to provide locally relevant evidence to support clinical decision-making and contribute to the optimization of thyroid management strategies in similar settings.

## METHODOLOGY

This retrospective cohort study was conducted at the department of endocrine and nuclear medicine of Northwest General Hospital and Research Centre. Patient data from January 2022 to December 2024 were included.

### Ethical Approval:

Data collection was started after obtaining approval from hospital IRB committee under Ref No. IRB&EC/2025-GH/0296; dated: September 29, 2025. The requirement for informed consent was waived by the IRB due to the retrospective design of the study and the use of de-identified patient data.

Patient demographics, clinical characteristics, treatment details, and hormonal data were extracted from the electronic medical records.

### Inclusion criteria:

Patients of either gender, age ≥18 years, diagnosed with Graves’ disease, toxic adenoma, or toxic multinodular goiter, who had undergone RAI treatment and and had complete clinical and laboratory data at both baseline and six-month follow-up. No cases with incomplete records or loss to follow-up were encountered during the study period. A total of 83 patients were included in the final analysis.

RAI therapy was administered using a fixed-dose protocol based on clinical diagnosis and disease severity. The administered dose ranged between 10–30 mCi, consistent with commonly adopted practices. Patients were prepared by discontinuing antithyroid drugs 5–7 days prior to therapy to enhance radioactive iodine uptake. A low-iodine diet was advised for approximately 1–2 weeks prior to treatment; however, adherence could not be objectively verified due to the retrospective nature of the study. Thyroid radioactive iodine uptake measurements were not routinely performed, as a fixed-dose regimen was employed.

Thyroid function was assessed at six months post-radioactive iodine (RAI) therapy, as most clinically significant hormonal changes and treatment outcomes are expected to manifest within the first 3–6 months, and early classification of thyroid status is commonly used in similar studies for initial outcome evaluation. Post-treatment thyroid status was classified based on biochemical parameters, using serum Thyroid-Stimulating Hormone (TSH) and Free T4 levels. Hypothyroidism was defined as elevated TSH above the reference range with low or normal Free T4 levels, euthyroidism as both TSH and Free T4 within the reference range, and persistent hyperthyroidism as suppressed TSH with elevated Free T4 levels.

Data were analyzed through SPSS-25. Descriptive statistics were used to summarize baseline variables. The normality of data was assessed through Shapiro Wilk test. Age was presented as means ± standard deviations. As the distribution of hormonal variables was non-normal, median (IQR) values were considered. Differences between pre- and post-treatment hormone values were assessed using the Wilcoxon Signed-Rank Test, given the non-parametric distribution of data. Associations between categorical variables such as post-treatment thyroid status (euthyroid, hypothyroid, hyperthyroid) and clinical/demographic factors (age group, gender, diagnosis, and RAI dose category) were determine through chi-square test or Fisher’s Exact Test. Due to small subgroup sizes and expected cell counts less than five in some comparisons, Fisher’s Exact Test was used where appropriate instead of the chi-square test to ensure validity of statistical inference. Binary logistic regression analysis was performed to identify predictors of post-treatment hypothyroidism (yes/no). Independent variables included age, gender, pre-treatment diagnosis, and RAI dose category. Adjusted odds ratios (ORs) with 95% confidence intervals (CIs) were reported.

## RESULTS

In this study a total of 83 Hyperthyroidism patients were included. The mean age of the patients was 49.76±15.2 years, ranged from 20 to 81 years, predominantly female 58 (69%). Most of the patients were aged between 40–60 years 35 (41.7%), followed by those under 40 years 26 (31.0%) and over 60 years 23 (27.4%). Graves’ disease was the most frequent diagnosis 46 (54.8%), followed by toxic adenoma 21 (25.0%) and toxic multinodular goiter 17 (20.2%).

Post-treatment, hypothyroidism was the most common outcome 37 (44.0%), while 35 patients (41.7%) became euthyroid and 12 patients (14.3%) remained hyperthyroid. Age and gender showed no significant association with post-treatment status (p = 0.060 and p = 0.364, respectively). Post-treatment thyroid status was significantly influenced by the presence of Graves’ disease (p < 0.001) and toxic adenoma (p < 0.001). Most patients with Graves’ disease developed hypothyroidism (71.7%), while those with toxic adenoma mostly achieved euthyroidism (90.5%). Toxic multinodular goiter was also significantly associated with post-treatment outcome (p = 0.011), with a higher proportion of euthyroid patients (64.7%) and fewer hypothyroid patients (11.8%) Table-I.

**Table-I:** Association of demographic and clinical characteristics with post-treatment thyroid status.

Variable	Category	Euthyroid n (%)	Hyperthyroid n (%)	Hypothyroid n (%)	p-value
Age	< 40 years	9 (34.6%)	2 (7.7%)	15 (57.7%)	0.060^1^
	40–60 years	16 (45.7%)	3 (8.6%)	16 (45.7%)	
	> 60 years	10 (43.5%)	7 (30.4%)	6 (26.1%)	
Gender	Female	27 (46.6%)	7 (12.1%)	24 (41.4%)	0.364^2^
	Male	8 (30.8%)	5 (19.2%)	13 (50.0%)	
Diagnosis	Graves’ disease	5 (10.9%)	8 (17.4%)	33 (71.7%)	<0.001^2^*
	Toxic adenoma	19 (90.5%)	0 (0.0%)	2 (9.5%)	<0.001^1^*
	Toxic multinodular goiter	11 (64.7%)	4 (23.5%)	2 (11.8%)	0.011^1^*

***Note:*** P value calculated through

1 Fisher’s Exact Test,

2 Chi-square test was used to calculate p-value.

* P-value <0.05 is significant.

A comparison of pre- and post-treatment thyroid hormone levels using the Wilcoxon Signed-Rank Test revealed statistically significant changes across all three markers. Median Free T4 levels decreased markedly from 2.10 to 1.00 ng/dl (Z = –7.835, p < 0.001), and T3 levels also showed a significant reduction from 1.90 to 1.00 ng/dl (Z = –7.857, p < 0.001), indicating effective suppression of thyroid hormone excess. In contrast, TSH levels increased significantly from 0.01 to 3.65 (Z = –7.807, p < 0.001), reflecting restoration of pituitary feedback following treatment. Overall, all hormonal changes were highly statistically significant (p < 0.001), demonstrating a strong biochemical response to RAI therapy. These hormonal changes reflect a significant therapeutic response to radioactive iodine therapy, with a substantial proportion of patients transitioning from a hyperthyroid to a hypothyroid or euthyroid state. This shift highlights the variability in post-treatment thyroid function and underscores the need for individualized dose titration and long-term monitoring to achieve optimal thyroid balance (Table-II).

**Table-II:** Pre- and Post-Treatment Hormone Levels.

Hormone	Time Point	Median (IQR)	Z	p-value
Free T4 (ng/dl)	Pre-RAI	2.10 (0.80)	–7.835	< 0.001*
	Post-RAI	1.00 (0.50)		
T3 (ng/dl)	Pre-RAI	1.90 (0.41)	–7.857	< 0.001*
	Post-RAI	1.00 (0.27)		
TSH (ng/dl)	Pre-RAI	0.01 (0.01)	–7.807	< 0.001*
	Post-RAI	3.65 (8.79)		

***Note:*** Wilcoxon signed-rank test was used to calculate p values;

* P value <0.05 is significant.

Binary logistic regression analysis was conducted to determine the independent predictors of post-radioiodine hypothyroidism. The overall model was statistically significant (χ^2^ = 39.62, p < 0.001), indicating that the predictors collectively contributed to the model. The model demonstrated good fit, as confirmed by a non-significant Hosmer–Lemeshow test (p = 0.461), and explained 50.4% of the variance in post-treatment hypothyroidism (Nagelkerke R^2^ = 0.504). The overall classification accuracy of the model was 78.6%, with 86.5% of hypothyroid cases and 72.3% of non-hypothyroid cases correctly classified. Among the variables examined, pre-treatment diagnosis showed a statistically significant association with post-radioiodine hypothyroidism. In particular, patients with Graves’ disease demonstrated higher odds of developing hypothyroidism compared to the reference category (adjusted OR = 16.48; 95% CI: 3.14–86.58; p = 0.001). However, the wide confidence interval indicates limited precision of this estimate, likely reflecting the sample size and distribution of outcomes; therefore, the magnitude of this association should be interpreted with caution. Age showed a borderline association with post-treatment hypothyroidism (adjusted OR = 0.962; p = 0.063), suggesting a possible trend toward lower odds with increasing age, although this did not reach statistical significance. No statistically significant associations were observed for gender (p = 0.815), toxic adenoma (p = 0.881), or RAI dose (p = 0.312) in the adjusted model (Table-III).

**Table-III:** Binary Logistic regression analysis of factors associated with post-rai hypothyroidism.

Variable	B	SE	Adjusted OR	95% CI	p-value
Age	-0.039	0.021	0.962	0.923 – 1.002	0.063
Gender (Male)	-0.149	0.636	0.862	0.248 – 2.998	0.815
Graves’ disease	2.802	0.846	16.48	3.14 – 86.58	0.001
Toxic adenoma	-0.167	1.114	0.846	0.095 – 7.51	0.881
RAI dose	0.092	0.091	1.097	0.917 – 1.312	0.312

***Note:*** Reference category for outcome: non-hypothyroid. Reference groups: Female (gender) and Toxic multinodular goiter (diagnosis). Wide confidence intervals may reflect limited sample size and should be interpreted with caution.

## DISCUSSION

This retrospective study examined the pre- and post-RAI hormone levels and clinical outcomes in patients with hyperthyroid diseases, with biochemical and clinical assessments six months after treatment. Among our patients, 44% became hypothyroid, 41.7% euthyroid and 14.3% hyperthyroid. Our results are in line with previously documented studies, such as Khan et al. (2023),^10^ who found a remission rate of 80.95%, and Pamnani et al. (2022),^11^ who reported 63.4% of patients developed hypothyroidism at six months. These findings indicate that the thyroid outcomes following RAI in our cohort are consistent with existing literature, and provide regional data from a tertiary care centre.

The changes in Free T4 (2.19 ng/dl to 1.01 ng/dl), T3 (2.01 ng/dl to 1.02 ng/dl) and TSH (0.68 ng/dl to 3.65 ng/dl), reflecting a decrease in T4 and T3 and an increase in TSH, reflect successful ablation of thyroid tissue and re-establishment of the hypothalamic-pituitary axis. These results are consistent with Anjum et al., 2025,^9^ and other reports detailing similar changes after RAI therapy.^12^,^13^ Clinically, these findings reflect the anticipated physiological response to treatment and do not describe a new effect, and confirm the expert opinion on post-RAI management.

In the current study, Graves’ disease was the predominant cause of hyperthyroidism and was more likely to develop hypothyroidism after RAI therapy. This finding is in line with other studies, including Akbarov et al. (2024),^13^ and could be potentially due to the diffuse nature of radioactive iodine uptake in autoimmune thyroid disease, which results in greater thyroid tissue damage. Conversely, patients with toxic adenoma were more likely to become euthyroid, which may be due to focal iodine uptake, and therefore, a more selective ablative effect.^14^ However, these findings should be interpreted with caution, as some of the subgroup analyses had a small sample size, which may affect the precision of estimates.

The association of RAI dose with treatment outcomes was assessed descriptively, though while some trends were noted between dose categories, and for different diagnostic subgroups, these should not be construed as representing a dose response. Some of the subgroups were small and the distribution of the data may be sparse. So, no conclusions can be made about dose effects from the current analysis.^15^-^17^

In the binary logistic regression, Graves’ disease was significantly associated with hypothyroidism following RAI. But the odds ratio (OR) is relatively large with a wide confidence interval, which indicates the estimate is not very precise, possibly due to the sample size and the distribution of outcomes. Age was not significantly associated in the final model. Similarly, gender and RAI dose were not associated with post-hypothyroidism.

### Limitations:

The small sample size, especially in some of the subgroups, may limit the statistical power and lead to less precise estimates. Low counts in certain categories may impact measures of association. The duration of follow-up was short (six months), which may miss long-term effects as hypothyroidism may occur months or years later following RAI therapy. In addition, the single-center retrospective nature of the study may affect its generalizability. The use of different biochemical cut-offs to define thyroid status may also add variability.^18^

However, this study offers valuable clinical insights into the post-RAI outcomes in a local cohort and may help inform patient advice and management strategies. Prospective studies with larger cohorts and longer follow-up are necessary to further assess predictors of response and long-term outcomes.

## CONCLUSION

In this cohort, hypothyroidism was a common outcome following RAI therapy, particularly among patients with Graves’ disease, while many with toxic adenoma achieved euthyroid status. Changes in thyroid hormone levels were consistent with expected post-treatment effects. Pre-treatment diagnosis was associated with outcomes in this cohort, however, findings should be interpreted cautiously due to small subgroup sizes. These results should be considered in light of the study’s limitations, including its retrospective single-center design, limited sample size, short follow-up, and lack of some relevant clinical variables. Overall, the study provides region-specific data and highlights the importance of appropriate follow-up after RAI therapy. Further prospective studies are needed to confirm these findings.

### Authors Contribution:

**AH:** Design, supervised, critically revised the manuscript.

**AN:** Data collection. Critical Review.

**MS:** Manuscript writing, responsible for integrity of research.

**BH:** Statistical analysis and wrote results.

All authors have read and approved the final version of the manuscript.
